# Connections, Communication and Collaboration in Healthcare’s Complex Adaptive Systems: Comment on "Using Complexity and Network Concepts to Inform Healthcare Knowledge Translation"

**DOI:** 10.15171/ijhpm.2017.138

**Published:** 2017-12-09

**Authors:** Tracey Bucknall, Danielle Hitch

**Affiliations:** ^1^School of Nursing and Midwifery, Faculty of Health, Deakin University, Geelong, VIC, Australia.; ^2^Alfred Health, Melbourne, VIC, Australia.; ^3^Occupational Therapy, School of Health and Social Development, Deakin University, Geelong, VIC, Australia.

**Keywords:** Complexity Theory, Complex Adaptive Systems, Clinical Decision Making, Systems Network Analysis, Integrated Knowledge Translation

## Abstract

A more sophisticated understanding of the unpredictable, disorderly and unstable aspects of healthcare organisations is developing in the knowledge translation (KT) literature. In an article published in this journal, Kitson et al introduced a new model for KT in healthcare based on complexity theory. The Knowledge Translation Complexity Network Model (KTCNM) provides a fresh perspective by making the complexity inherent in complex systems overt. The model encourages a whole system view and focuses on the interdependent relationships between actions, interactions and actors. Taking a systems approach assists our understanding of the connections, communication and collaboration necessary to promote knowledge mobilisation and facilitate the adoption of change. With further development, this could enable the targeting of more effective strategies across the various stakeholders and levels of service, fostering redesign and innovation.

In the complex, dynamic nature of healthcare today, increased access to synthesised evidence to inform clinical decisions, and demands for improved clinical and cost effectiveness, add considerable pressure to the clinical environment. In 2010, research indicated that 75 clinical trials and 11 systematic reviews were published each day.^1^ Not surprisingly then, a clinician or decision maker’s ability to synthesise large volumes of information, adapt and apply it into varying contexts has been found wanting. Several studies have revealed large gaps in care between what patients *should* receive and what they *actually* receive in practice.^2,3^

The translation of evidence to inform real world practice suggests such variation is rectifiable by simply communicating research to clinicians. However, growing evidence of the low to moderate success of single, and particularly multiple, interventions in changing clinician behaviour suggests this strategy is based on two assumptions – better tools result in better outcomes and clinicians will respond to better evidence. As a result, there have been numerous calls for new paradigms, including widespread advocacy for the adoption of complexity theory. In this commentary, we briefly discuss complexity science and the fit with KT activities in health care. We will discuss how the KT Complexity Network Model^4^ (KTCNM) proposed by Kitson and colleagues provides a fresh perspective on KT in relation to other existing models, and lastly, we will consider the strengths and limitations of the models’ theoretical underpinnings as reported by Kitson et al.

Complexity science provides a framework for understanding practice change.^4-6^ It offers the potential to explore how systems actually behave, rather than how they should behave, and to learn about sustainability and adaptability as change occurs. Complexity science assists us to examine the erratic, uncontrollable and unbalanced aspects of organisations such as healthcare organisations.^7^ Literature on organizational change has been known to give the impression of a rational, controlled, and orderly process. Yet in practice, it is often chaotic and non-linear with unexpected outcomes.^7^

Health services research often focuses on microsystems and single disciplines when implementing practice change. However, this reductionist view does not provide an understanding of how elements are organised, the extensive interactions that occur, nor the feedback mechanisms that accelerate spread and adoption. Complex systems thinking recognises that systems are made of a series of connected elements, which collectively form the whole. Complex systems are not chains of linear cause and effect relationships, but rather networks of interrelationships. Each element has an influence on other elements, and may act in an unpredictable way.^8^ This influence may result in positive or negative feedback for other elements, contributing to an emerging and evolving system.

In contrast to linear systems, where the strength of the response is proportionate to the strength of the stimuli, in non-linear systems proportionality does not hold: small changes can have large and unanticipated results.^9^ Similarly, large changes can have a negligible effect. A key feature of a complex system is the principle of self-organisation, which states that organisations are influenced by both positive and negative feedback loops.^10^

Complex adaptive systems (CASs) are those capable of self-organisation, where patterns reproduce and the organisation simultaneously adapts, responds and contributes to change.^6^ If we believe that health care organisations are CASs, functioning in a non-linear way, capable of self-organisation and evolution, then it seems apparent that KT models and frameworks should accommodate self-organisation. As Kitson et al^4^ and others^5,7^ have identified, the concept of CASs offers new possibilities for understanding change within a healthcare organisation.

Effective decision-making needs to acknowledge unpredictability and build on subtle emergent forces. Peirce contends that traditional organizational models fail to incorporate the experience and learning that creates shifting adaptability.^11^ Nor do they deal with paradox, uncertainty, disagreement and surprise. Yet, when individuals are confused, uncertain and disagree, creativity may emerge, and self-organisation can occur.^11^ Examining CASs requires complex systems to be studied from diverse perspectives across different levels.^10^ These diverse perspectives are required to confront the paradoxes within healthcare, reframe our understandings and unravel its intricacies.^11^ Increasingly, these perspectives have been incorporated into KT theoretical frameworks that have emerged in the past two decades.

The use of complexity and networking theory in the KTCNM is both complementary to, and a departure from, the existing theoretical frameworks in KT. Through their work on Collaborations for Leadership in Applied Health Research and Care (CLAHRC) in the United Kingdom, Lockett et al^12^ identified five archetypes used to organise KT in healthcare. This study of nine CLAHRCs used a longitudinal mixed methods approach that incorporated case studies, social network analysis, field observations and qualitative interviews over a four year period. Each archetype describes a different organisational structure including: those which enable multidisciplinary research processes; loosely autonomous research streams with identified knowledge brokers; independent or modular research and KT activities; loose collaborative networks, and; centrally organised service improvement programs.^12^

The archetypes represent general ways of organising KT activities within specific organisational contexts, and the authors acknowledge that they are often applied in a hybrid form in the real world. No particular archetype is proposed to be more effective than any other, and their development was an attempt to simply describe current practice. The KTCNM is an example of Archetype A, that is a framework that promotes the purposeful integration of multi-sectorial stakeholders into research and implementation processes. Arguably, the KTCNM is more broadly inclusive of stakeholder groups with its inclusion of the community, education and government sectors.

Nilsen^13^ proposed a taxonomy of implementation theories, models and frameworks, which included five theoretical approaches which addressed three overarching implementation aims. The five theoretical approaches were: process models; determinant frameworks; classic theories, implementation theories and evaluation frameworks. The aims were to (1) describe and/or guide the process of KT, (2) understand and/or explain influences on KT outcomes, and (3) evaluate implementation. The KTCNM is distinctive from other KT theoretical frameworks in its attempt to simultaneously address two implementation aims. One the one hand, the KTCNM operates as a process model, with five clusters identified as the key areas for processes and tasks which enable KT.^4^ These clusters describe (and could be used to guide) the KT process. On the other hand, it operates as an implementation framework to explain the influences on KT outcomes.

Some KT process models (particularly those using an evidence based practice lens) adopt a linear approach to KT tasks, such as the ACE Star Model of KT^14^ and the Quality Implementation Framework.^15^ However, several others are more reflective of complex systems thinking in their adoption of interactive and dynamic representations of the tasks and stakeholders involved in KT, such as the Ottawa Model of Research Use.^16^ One of the best-known process models is the Knowledge to Action Framework,^17^ which is based on two interdependent components (knowledge creation and the action cycle), that interact iteratively over time.^18^ In their description of the KTCNM, Kitson et al^4^ have more explicitly described how clusters interact dynamically in time and space as part of CASs. By visually depicting the model in a non-linear format, KTCNM highlights the different level of influence and focus given to each cluster at various points in time, and their on-going mutual interactions. This allows the KTCNM to communicate the inherent complexity on which it is predicated more successfully.

However, the KTCNM also operates as an implementation theory by providing an understanding of important factors and aspects (in this case, the complex system networks associated with KT) that may be influential to KT outcomes. This differentiates it from determinant models, such as the Promoting Action on Research Implementation in Health Services (PARIHS)^19^ and the Consolidated Framework for Implementation Research (CFIR),^20^ which specify individual determinants that may act as barriers or facilitators for KT.^13^ A focus on relationships, between individuals, organisations and sectors, is the unique feature of the KTCNM. While previous theories have identified stakeholders involved in KT, to date little theoretical development has been undertaken by Kitson and colleagues into how the relationships between them, across multiple levels, actually operate in healthcare. By making the often unpredictable and complex nature of these interactions overt, the KTCNM supports a better understanding of how the KT process (with its often assumed linearity) can so often be subverted in real life. However, these propositions are yet to be tested.

In regards to the third aim of KT theories - the evaluation of implementation - Kitson et al^4^ propose the KTCNM as a future avenue for inquiry to understand how clusters self-organise and evolve. However, at this point these lines of inquiry, and the ways in which to pursue them, are yet to be clearly identified. Given the emphasis on relationships in the KTCNM, the use of social network analysis could potentially be used to evaluate KTCNM. Social network analysis is an emerging method that aims to visualise and analyse complex interrelationships from a network theory perspective. This form of analysis looks at both the relationships between individuals (or groups, or organisations), and the resources that these relationships facilitate and, at the meta level, how individual behaviour relates to systemic change. Few projects to date have used social network analysis to explore complexity in healthcare KT,^21,22^ although those that have report it offers considerable insight. It was also one of the methods used to develop the archetypes previously mentioned,^12^ where this analysis was undertaken longitudinally as part of a multiple methods program of inquiry.

In considering the strengths and limitations of the KTCNM at this point in time, the model offers a description of the properties. It is well described and coherent, and it enables comparison of the properties with other theories. It does not claim to be explanatory in nature, specifying causal relationships and mechanisms of implementation. It is not yet predictive of relationships between the properties nor does it hypothesise.

In moving forward with the theoretical development of KTCNM, there are lessons from research using complexity theory in healthcare that Kitson and colleagues could address. To date, it has highlighted a lack of conceptual clarity and inconsistent use of principles.^23,24^ Although Thompson et al^24^ found an increasing number of studies examining relationships and interactions, the findings were hampered by varying definitions of complexity and inconsistent incorporation of complexity theory. Moreover, Brainard et al’s^25^ review examined the efficacy of 22 interventions informed by complexity theory. However, they were unable to establish effectiveness due to the way the interventions were designed and impacts reported. In the absence of conceptual clarity, our insight into the impact of complexity theory will be limited. This risk was highlighted by Braithwaite et al,^5^ who also appeal for a move from descriptive to more explanatory interventional research in order to advance our understanding.

## Conclusion

Overall, the KTCNM offers a helicopter view of the actions, interactions and actors previously addressed infrequently and inconsistently in KT research. The helicopter view reminds us to move away from a reductionist, micro examination of individuals and settings to viewing the context as a whole, seeing both interactions and their consequences. As advocated by Kitson and colleagues, we should move beyond traditional mechanistic, linear approaches. We need to focus more on understanding the communication and collaboration required between individuals, disciplines, organisations and systems to improve knowledge mobilisation and adoption of change. In health services, hierarchies are often entrenched in governance structures and disciplinary silos. Overcoming such power imbalances and disciplinary cultural divides is no simple matter. As such, research should not only focus on the instillation of knowledge or interventions, but also emphasise the interrelationships and outcomes that have made them happen (or not) in capricious environments.^23^

At this stage, no single model addresses the three aims of implementation science. However, the CAS concept advocated by Kitson and colleagues offers new possibilities for understanding change within healthcare. It can assist us to examine the unpredictable, disorderly and unstable aspects of healthcare organizations, to enable the success of initiatives which improve patient outcomes, increase adherence to practice changes, and develop new understandings of strategies to foster sustainable systems. A natural progression from understanding how something works, to encompass other key questions (such as who we need to work with, when we need to work with them), may assist our ability to target more effective strategies across the various stakeholders and levels of service.

## Ethical issues

Not applicable.

## Competing interests

Tracey Bucknall collaborates with Gill Harvey in research.

## Authors’ contributions

Both authors contributed to the conceptualisation of the paper, drafting and agreement on the final paper.

## Authors’ affiliations

^1^School of Nursing and Midwifery, Faculty of Health, Deakin University, Geelong, VIC, Australia. ^2^Alfred Health, Melbourne, VIC, Australia. ^3^Occupational Therapy, School of Health and Social Development, Deakin University, Geelong, VIC, Australia.
